# Far-field radially polarized focal spot from plasmonic spiral structure combined with central aperture antenna

**DOI:** 10.1038/srep23751

**Published:** 2016-03-24

**Authors:** Lei Mao, Yuan Ren, Yonghua Lu, Xinrui Lei, Kang Jiang, Kuanguo Li, Yong Wang, Chenjing Cui, Xiaolei Wen, Pei Wang

**Affiliations:** 1 Department of Optics and Optical Engineering & Anhui Key Laboratory of Optoelectronic Science and Technology, University of Science and Technology of China, Hefei, Anhui, 230026, China; 2 Center for Micro- and Nanoscale Research and Fabrication, Hefei National Laboratory for Physical Sciences at the Microscale, University of Science and Technology of China, Hefei, Anhui 230026, China

## Abstract

Manipulation of a vector micro-beam with an optical antenna has significant potentials for nano-optical technology applications including bio-optics, optical fabrication, and quantum information processing. We have designed and demonstrated a central aperture antenna within an Archimedean spiral that extracts the bonding plasmonic field from a surface to produce a new vector focal spot in far-field. The properties of this vector focal field are revealed by confocal microscopy and theoretical simulations. The pattern, polarization and phase of the focal field are determined by the incident light and by the chirality of the Archimedean spiral. For incident light with right-handed circular polarization, the left-handed spiral (one-order chirality) outputs a micro-radially polarized focal field. Our results reveal the relationship between the near-field and far-field distributions of the plasmonic spiral structure, and the structure has the potential to lead to advances in diverse applications such as plasmonic lenses, near-field angular momentum detection, and optical tweezers.

Structured light has become a significant research topic over the last two decades^1^,^2^. Because of its unique phase or polarization distributions, structured light has been widely applied in a number of fields, including quantum communications^3^,^4^, optical manipulation^5^,^6^,^7^, and super-resolution imaging^8^,^9^,^10^. As the requirements for these micro/nano-scale optical elements increase, more and more nanostructures, such as plasmonic lenses and metasurfaces, are being designed and fabricated to generate unique phase and intensity distributions in the near or far fields^11^,^12^,^13^,^14^. As a typical plasmonic lens, the bull’s-eye structure has been studied thoroughly over many years because of properties that include focusing of surface plasmons (SPs) and field enhancement^15^. By adding some different antenna structures at the centre of the structure, the highly localized focal field of the plasmonic lens can be narrowed further down to the subwavelength scale, which can be used in super-resolution lithography^16^,^17^ or as an ultra-compact plasmonic spectral-band demultiplexer for telecommunications^18^. In addition, discrete components arrayed in a circle can also be used as a plasmonic lens to improve the excitation efficiency or to provide unique polarization properties^19^,^20^,^21^.

Radially polarized incident light is generally used to excite SPs efficiently on the bull’s-eye plasmonic lens, which introduces an alignment problem because the centre of the radially polarized light must be aimed at the centre of the bull’s-eye to ensure constructive interference at the focal spot. To overcome this shortcoming, the plasmonic Archimedean spiral lens (PASL) has been developed^22^. The chirality of the spiral provides a new degree of freedom to manipulate not only the intensity distribution but also the spin or orbital angular momentum of the light. In contrast to the bull’s-eye lens, circularly polarized light is recommended to excite the SPs of the PASL, and this polarization is much easier to realize^23^. In fact, the Archimedean spiral structure is also regarded as a circular polarization analyzer because different focal SP field distributions are produced under excitation by light with right-handed circular polarization (RCP) and left-handed circular polarization (LCP)^24^. This unique polarization-dependent characteristic can also be applied in near-field optical manipulation^25^,^26^ and vortex phase generation applications^27^,^28^,^29^. Because SPs decay exponentially with the distance away from the surface, the reported focal field of the PASL is always restricted to within the near field, which limits its practical application. With the help of the central antenna, far-field beams with tunable vortex phases can be tailored using spiral nanostructure designs^30^,^31^. However, the relationship between the near-field and far-field distributions of the spiral structure has scarcely been discussed, and the polarization of the far-field in different propagation positions has also rarely been studied.

In this paper, we milled a rectangular hole in the centre of an Archimedean spiral, which acts as a scattering antenna, to pull the focal plasmonic field away from the sample surface. The far-field focal field of the holed-PASL was observed by three-dimensional confocal microscopy. The field distributions in the near and far fields, which are affected by the interactions between the chirality of the Archimedean spiral and the spin angular momentum of the incident light, were analysed. A far-field radially polarized focal electromagnetic field can be generated in the case of a left-handed spiral excited by RCP light. The focal vector field was predicted theoretically based on Huygens ’ Principle and plasmonic field excitation theory, and the results agreed well with those of the experimental observations.

## Results

### Structure design and theoretical predictions

**Figure 1:**
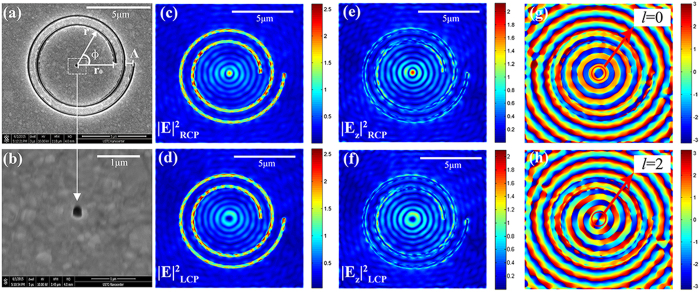
Images and simulations of the plasmonic Archimedean spiral lens. Scanning electron microscope images of (**a**) the spiral structure and (**b**) the central antenna. The scale bars in the pictures represent 5 μm and 1 μm, respectively. (**c**–**h**) Near-field intensity and phase distributions simulated at incident wavelength of 532nm by the finite-difference time-domain (FDTD) method. Electrical field intensity |E|^2^ distributions on the surface of the structure under illumination with (**c**) right-handed circular polarization and (**d**) left-handed circular polarization. (**e**,**f**) Corresponding |E_z_|^2^ intensity distributions that reveal that the main component of the near-field is polarized in the z-direction. (**g**,**h**) Corresponding phase distributions. RCP illumination will create a near-field distribution that carries no vortex phase. A vortex phase with a topological charge of 2 will be generated by LCP excitation.

As shown in Fig. 1a (the structural parameters are presented in the Methods section), a PASL can be mathematically described in cylindrical coordinates (r, *ϕ*) as


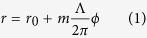


where *r*_0_ is the starting radius constant, Λ is the thread pitch of the spiral, which is designed to be equal to the wavelength of the SP *λ*_*SPP*_, and m is an integer that represents the screw numbers. As a plasmonic lens, the intensity distributions of the SP field generated by the plasmonic Archimedean spiral, which depend on the handedness of the incident circularly polarized light, have been experimentally verified by NSOM (near-field scanning optical microscopy) in earlier study^24^. To transfer the SP field to the far field, a tiny rectangular hole (108.3 nm by 128.7 nm) is milled at the centre of the spiral to function as a scattering antenna (Fig. 1b). The optical properties of the holed-PASL are determined synthetically by the plasmonic spiral and the scattering antenna.

First, the near-field electromagnetic field of the PASL was investigated via numerical simulations. Figure 1c,d show the near-field distribution of a left-handed PASL under right-handed and left-handed circular polarization excitation, respectively. The central electromagnetic fields mainly arise from the SPs that are concentrated by the spiral slits. Comparison of the total field intensity |E|^2^ (Fig. 1c,d) and the z-component intensity |E_z_|^2^ (Fig. 1e,f) indicates the central electromagnetic field is dominated by the z-polarized component, which can be expressed in cylindrical coordinates (R, *θ*) as follows^32^:


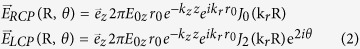


where 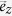 is the direction vector, *E*_0*z*_ is the amplitude constant in the z-direction, and 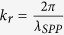 represents the wave vector in the sample plane. Here we notice that there is a vortex phase item, e^2*iθ*^ for 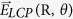 with topological charge of *l* = 2, but no corresponding item exists in 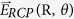, which is read from the phase distribution pictures of Fig. 1g,h.

The interactions between the light and the PASL comply with the law of conservation of angular momentum^33^,


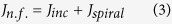


where *J*_*n.f*_ , *J*_inc_, *J*_spiral_ represent the angular momentum of the near-field electromagnetic field, the incident light and the spiral structure, respectively. Using our experiment as an example, *J*_spiral_ is +1, *J*_inc_ is −1 for the right-handed circular polarization and +1 for the left-handed circular polarization. As a result, *J*_*n.f.*_ will naturally be equal to 0 for RCP excitation and 2 for LCP excitation.

**Figure 2:**
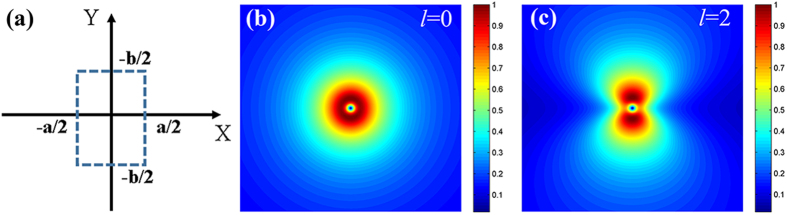
Sketch diagram of the dipole superposition model. (**a**) The coordinates used in the dipole superposition model. The dipoles were placed around the rectangular antenna (the dashed line) in the x-y plane where z = 0. We selected the side lengths of the central antenna (a = 108 nm and b = 128 nm) and the observation plane was placed at z = 5 μm. Differently polarized excitations will generate different phase distributions, which then lead to the different initial phases for these dipoles. The two pictures on the right are the far-field patterns of the dipole array (**b**) without the vortex phase and (**c**) with the vortex phase of *l* = 2.

For the holed-PASL, the focused SP field (Fig. 1) that is concentrated into the central antenna will generate a series of z-direction dipoles around the rectangular central antenna. Clearly, different near-field distribution will generate different far-field distribution during the scattering process. And the far-field electromagnetic distribution of the focal spot can then be regarded as the integral of the radiation of these dipoles, according to Huygens’ Principle. The electrical field of the single dipole radiation in the far field can be described as follows^34^:


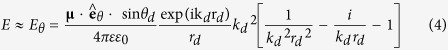


where **μ** is the electric dipole moment, which is proportional to the localized excitation field described by Equation 2 for the holed-PASL (see Fig. 1). It should be noted that although the electric field **E** actually consists of two components in the radial and azimuthal directions, the E_r_ component of the dipole radiation, which mainly contains near-field items, can be neglected for our observation distance (~z = 5 μm). The subscript *d* is used to indicate “dipole” parameters here to distinguish them from the previous parameters. On this basis, we add the initial phase item to the dipole array in the near-field. The initial phases of the dipoles are decided by the incident light polarization. When the left-handed PASL is illuminated with RCP light, the dipole array carries no vortex phase (*J*_*n.f.*_ = 0) and the array will form a doughnut pattern in the far-field, as shown in Fig. 2b. The pattern is similar to that for the single dipole radiation because the size of the central antenna (~100 nm) is much smaller than the distance between the sample and our observation plane (~z = 5 μm). In contrast, when the left-handed PASL is illuminated with LCP light, the dipole array will carry an initial vortex phase of *l* = 2 and will create a butterfly-like (two-lobe) pattern in the far-field, as shown in Fig. 2c. The phenomenon of intensity division in one direction is mainly caused by the asymmetries of the central antenna and the initial vortex phase. This means even when it is illuminated by a plane wave, an asymmetric rectangular aperture antenna will generate a slightly elongated intensity pattern. Moreover, the vortex phase breaks the symmetry of the initial phase, which leads to a difference between the far fields of the two circular polarizations, and this can be used in near-field angular momentum detection research. In addition, the electromagnetic field pattern depends strongly on the topological charge of the vortex (see Supplementary Materials, Figure S1).

### Characteristics of three-dimensional focal fields

**Figure 3:**
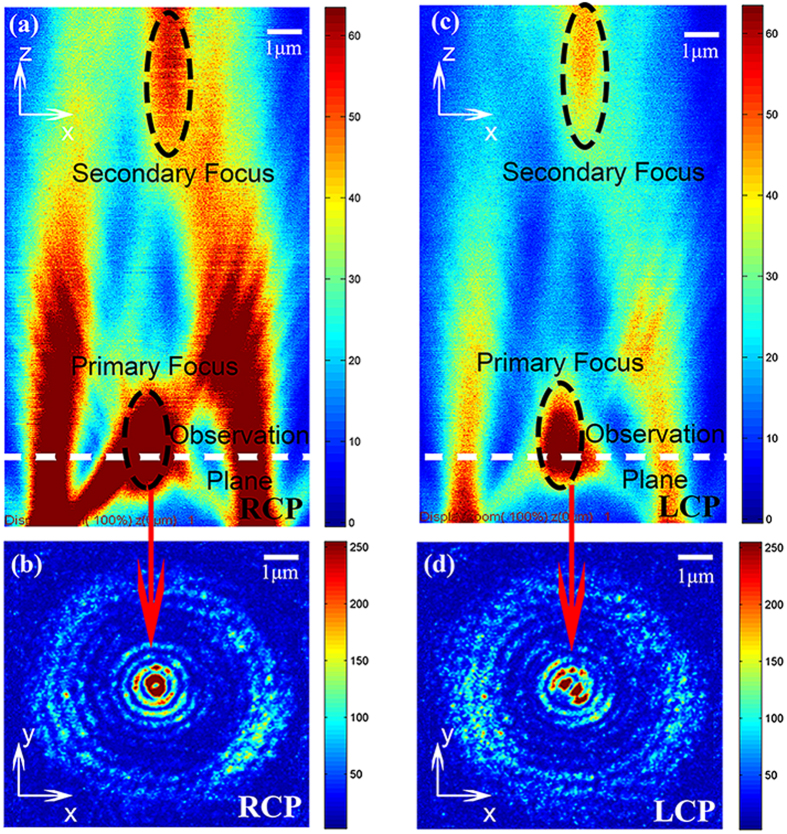
Transmission field characteristics. Experimental intensity distributions of the transmission field of the PASL structure are shown under (**a**) RCP and (**c**) LCP illumination, pictured via a Z-depth scan module (9 × 15 μm^2^). The bottom of the picture is the sample plane and the observation plane is indicated in the pictures by white dashed lines. Every altitude (Z value) in (**a,c**) represents an observation plane. The white dashed lines in (**a**,**c**) are cross-sections of those figures shown in (**b,d**), representing selected observation planes. Two focal spots has been marked in black dashed circles. Experimental images of the first primary focal spots in the observation plane are also shown under conditions of (**b**) RCP and (**d**) LCP illumination.

Next, the three-dimensional focal fields are characterized via confocal microscopy (Olympus IX71 FV300). Figure 3 depicts both the longitudinal (see Fig. 3a,c) and transverse (see Fig. 3b,d) field distributions of the holed-PASL when excited by either right-handed (see Fig. 3a,b) or left-handed (see Fig. 3c,d) circularly polarized laser light (λ = 532 nm). The far-field focal plane is easily determined in the Z-depth scanning pictures (see Fig. 3a,c) to be 2 μm away from the sample surface. It is clearly shown in Fig. 3a,c that there are two focal spots in the transmission field. Notably, when we repeat the experiments at different incident wavelengths (λ = 633 nm), the longer wavelength brings the secondary focal spot nearer while the position of the primary focal spot remains almost unchanged (see supplementary Figure S2). This means that the central antenna plays a dominant role in the first primary focal plane and that the secondary focal spot in the far field has mainly arisen from the diffracted field of the spiral slits. In addition, as shown in Fig. 3b,d, RCP laser light will generate hollow ring intensity distributions in the observation plane, while LCP laser light produces a focal field with two lobes, and these results are in good agreement with the analytical results above (see Fig. 2b,c). The far-field focal patterns are quite different from the calculated near-field distributions shown in Fig. 1c (a solid dot) and 1d (a hollow ring). This difference simply reveals the crucial role of the central antenna in producing the far-field focal spot of the plasmonic lens. As it suffers from bounding characteristics in the surface, the near-field focal field of the normal plasmonic lens is difficult to be measured directly, because the NSOM probe perturbation makes the measured picture quite different to the actual picture in the near field. In our device, the scattering aperture antenna at the centre of the spiral structure pulls the near-field focal spot generated by the PASL out to the far field. This function will not only facilitate the near-field vortex phase measurement but also ease optical tweezers applications by keeping the disturbance caused by the surface resistance of the substrate away^35^.

**Figure 4:**
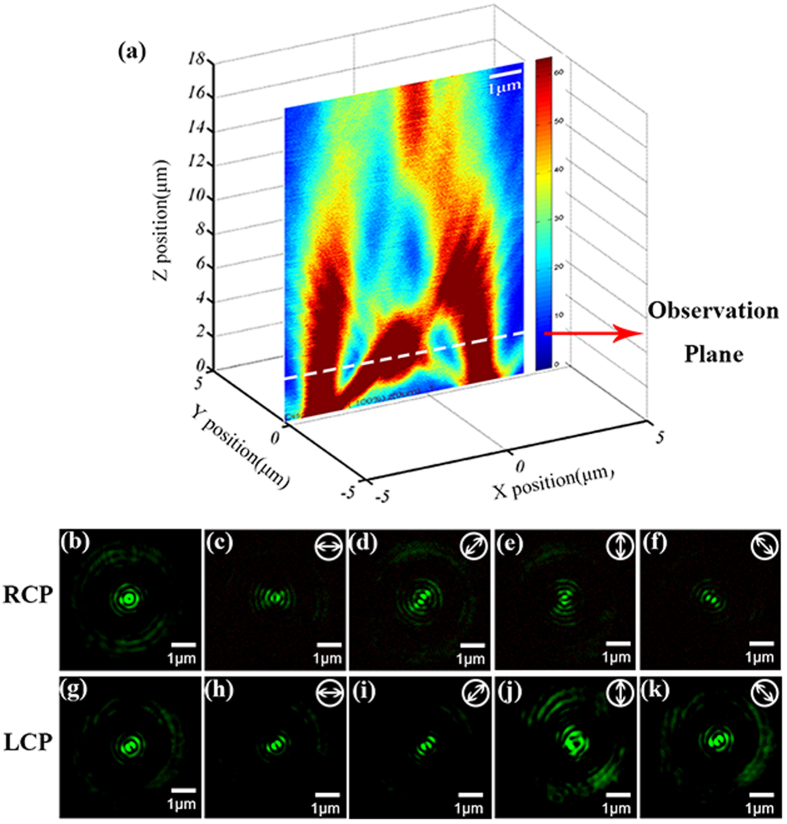
Intensity distributions for the two different circular polarization excitation cases. (**a**) The observation plane was placed in the first focal plane by the Z-depth scan module. The intensity distributions are shown under (**b**) RCP and (**g**) LCP conditions without the analyser. The intensity distributions are then shown in the (**c**–**f**) RCP and (**h**–**k**) LCP cases with the analyser. The polarization orientation of the analyser is depicted by the white arrows in the top-right corner of each picture.

The polarization of the focal spot in the far field is also investigated in our experiments. It is known that radially polarized light can generate a focal field that can be considered to be an imperfect vertical electric dipole without near-field items^36^. According to the principle of reversibility of light, a vertical electric dipole will naturally also produce radially polarized light in the far field. In our specific structure, the spiral will act as a z-dipole source provider. The concentrating SP that originated from the spiral excites the z-direction dipoles around the central hole. Also, the central aperture antenna can be regarded as an emitter. Under RCP excitation, all the emitter dipoles are in-phase, and this is equivalent to a larger vertical electric dipole. Therefore, the focal field should be radially polarized. Then we experimentally checked the polarization at the focal plane, as indicated in Fig. 4a, and the results are presented in Fig. 4b–f. Figure 4b shows the intensity pattern without the analyzer, which has a doughnut shape, and Fig. 4c–f show that the extinction directions of the intensity distributions are rotating along with the analyzer’s orientation. This clearly signifies that the first primary focal spot is now radially polarized. Because of the vortex phase of the near field, the total polarization of the dipoles that are arrayed around the aperture antenna cannot be equivalent to a single z-dipole. Therefore, the intensity distributions do not change with the analyzer in the LCP excitation case (see Fig. 4h–k), which indicates the circular polarization of the focal field. We also check the polarization state of the secondary focal spot that arises from the diffraction of the spiral, and it is always circularly polarized, regardless of whether RCP or LCP excitation is used (see supplementary Figure S3).

## Discussion

**Figure 5:**
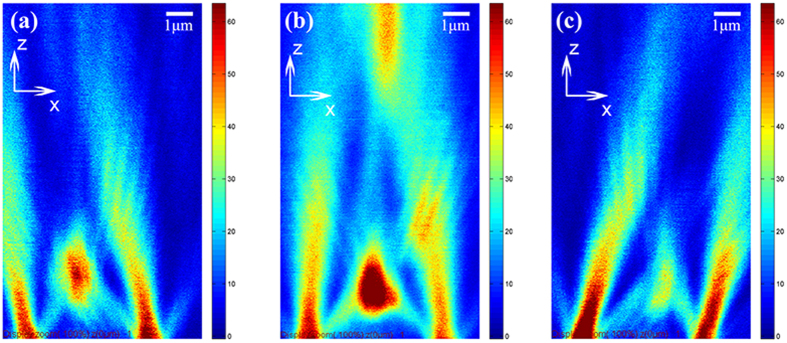
Scanned images of the transmission field. (**a**–**c**) The focal spot position can be modulated by controlling the angle of incidence to provide a radially polarized scanning optical tip near the sample plane.

Radially polarized light has been widely used in optical trapping applications^37^. The flexibility of the PASL’s transmission field also suggests great potential for application to optical manipulation. For example, switchable polarizations can be obtained by adjusting the distance in the z-direction; by controlling the angle of incidence, the focal spot position can be scanned in the longitudinal plane (see Fig. 5) to realize dynamic manipulation.

As mentioned earlier, the chirality of the structure will interact with the angular momentum of incident light. Here we provide more results for the dipole array with different vortex phases, as shown in Figure S1 in the supplementary material. These results indicate that if the incident light carries more complex angular momentum information, some different intensity distributions may be created. As we know, the spin angular momentum of the light corresponds to the chirality of the circular polarization, which only has three possible states (±1, 0). To acquire more angular momentum information, the orbital angular momentum, which is related to the vortex phase of the light, should be introduced. However, the incident vortex phase will dramatically affect the initial phase of the secondary wave source on the spiral. As a result, it is difficult to obtain a stable and clear image without settling the perfect alignment problem. After all, the central antenna in the structure offers the possibility of advances in near-field vortex phase detection.

In conclusion, we have designed and demonstrated a plasmonic Archimedean spiral lens with a central antenna. Unlike a traditional plasmonic lens, which generates a near-field distribution, the central antenna here extracts the bonding SPP field from the surface to produce a vector focal field in far field. By modulating the chirality of the incident circular polarization, the left-handed spiral (one-order chirality) can output micro-radially and circularly polarized focal fields. We therefore believe that the structure and concepts discussed here have fundamental importance for both plasmonics and nanophotonics, and provide a basis for novel applications such as optical tweezers and polarization and angular momentum detectors.

## Methods

### Sample preparation

The PASL was fabricated by focused ion beam milling (FIB, FEI Helios Nano Lab 650 System) technology on a 50-nm-thick silver film that had been evaporated on a glass substrate (see Fig. 1a,b). The gap width of the spiral is approximately 117 nm, which is much smaller than the wavelength of the SPs along the silver film.

### Experimental setup

**Figure 6:**
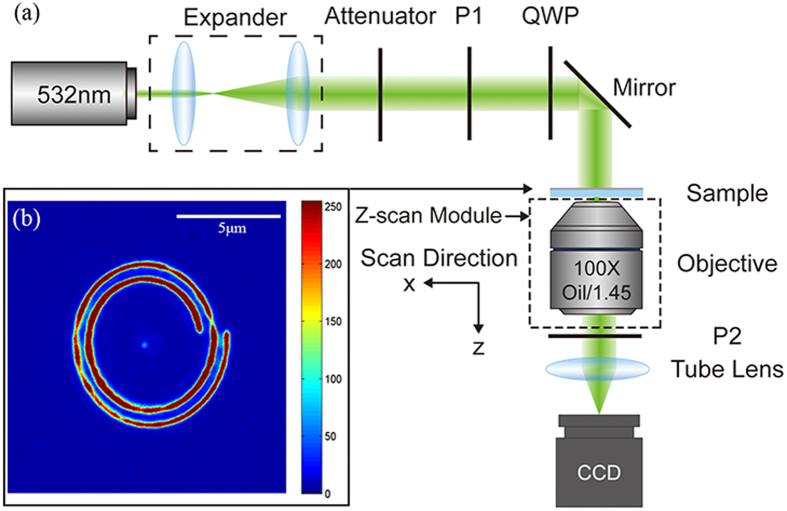
Schematics of the experimental setup. (**a**) The coordinates in the picture show the direction of the Z-depth scan module of the microscopy system. (**b**) Image of the spiral structure under white light illumination. The thread pitch of the spiral is Λ = 508.9 nm.

The focal optical field of the holed-PASL device was measured in three dimensions using a confocal microscope (see Fig. 6a). A linearly polarized TEM_00_ laser beam (wavelength of 532 nm; 10 mW laser power) propagates through an expander lens system to obtain the desired beam radius, and then passes through an attenuator, a polarizer and a quarter-wavelength plate to form the desired circularly polarized light before finally illuminating the sample normally via a mirror. By rotating the quarter-wavelength plate, the chirality of illuminating light can be easily controlled. A 100× oil-immersion objective (numerical aperture of 1.45) underneath the sample is used to collect the transmitted light and image the focal spot, when used together with a tube lens. The focal field polarization is checked by the analyzer, which was inserted before the charge-coupled device (CCD) detector. The device is placed on a confocal microscope to image the focal spot in three dimensions. Figure 3b shows experimentally acquired images of the spiral structure under white light illumination. With the help of these white light images, we can easily adjust the angle and position of incidence. The transmitted field distribution of the left-handed PASL in the X-Z plane is captured by the Z-depth scan module of the Olympus IX71 confocal microscopy system.

## Additional Information

**How to cite this article**: Mao, L. *et al*. Far-field radially polarized focal spot from plasmonic spiral structure combined with central aperture antenna. *Sci. Rep.* **6**, 23751; doi: 10.1038/srep23751 (2016).

## Supplementary Material

Supplementary Information
