# Supplementary material for: Far-field radially polarized focal spot from plasmonic spiral structure combined with central aperture antenna
Source: Sci Rep. 2016 Mar 24;6:23751. doi: 10.1038/srep23751 (PMC4806319; doi:10.1038/srep23751)
Supplement: Supplementary Information [file srep23751-s1.pdf]

Supplementary Materials For

Far-field radially polarized focal spot from plasmonic spiral structure combined with central aperture antenna

Lei Mao[1], Yuan Ren[1], Yonghua Lu[1]*, Xinrui Lei[1], Kang Jiang[1], Kuanguo Li[1], Yong Wang[1], Chenjing Cui[1], Xiaolei Wen[2] and Pei Wang[1]*

[1]Department of Optics and Optical Engineering & Anhui Key Laboratory of Optoelectronic Science and Technology, University of Science and Technology of China, Hefei, Anhui,230026, China
[2]Center for Micro- and Nanoscale Research and Fabrication, Hefei National Laboratory for Physical Sciences at the Microscale, University of Science and Technology of China, Hefei, Anhui 230026, China

Correspondence and requests for materials should be addressed to Yonghua Lu (e-mail: yhlu@ustc.edu.cn) or Pei Wang (e-mail: wangpei@ustc.edu.cn)

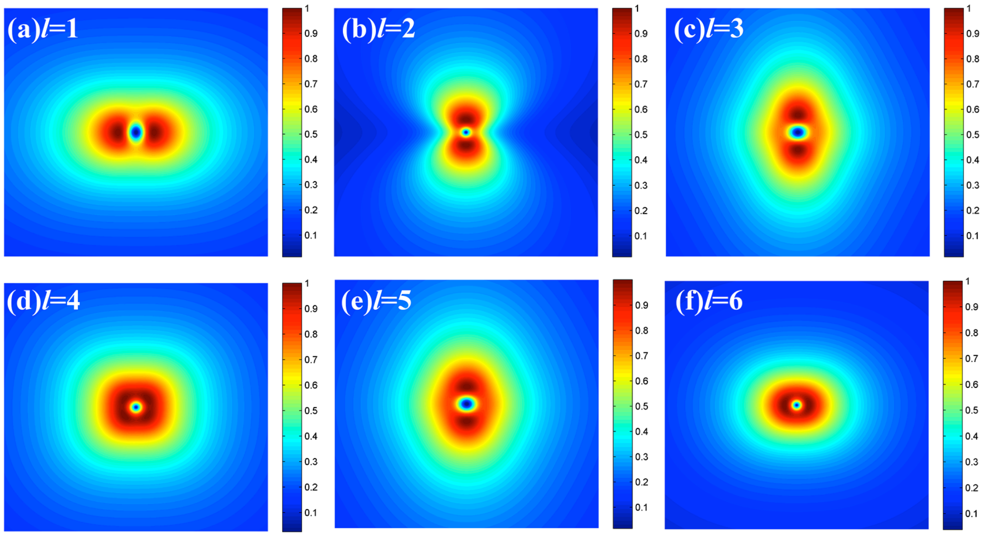

Supplementary Figure S1 | Far-field patterns of the dipole array with different vortex phases. (a)–(f) Topological charges of the vortex phase for *l* =1–6.

As mentioned in the main text, when we repeat the experiment using a different incident wavelength (λ = 633 nm), the longer wavelength brings the secondary focal

spot nearer, while the primary focal spot's position remains almost unchanged (see Figure S2). This is because longer wavelengths will lead to wider diffraction angles for the same slot widths, so that the overlapping point moves closer. We can therefore draw the conclusion that the secondary focal spot is formed by interference between the spiral slots on both sides, while the primary focal spot is mainly composed of the scattered field from the centred antenna.

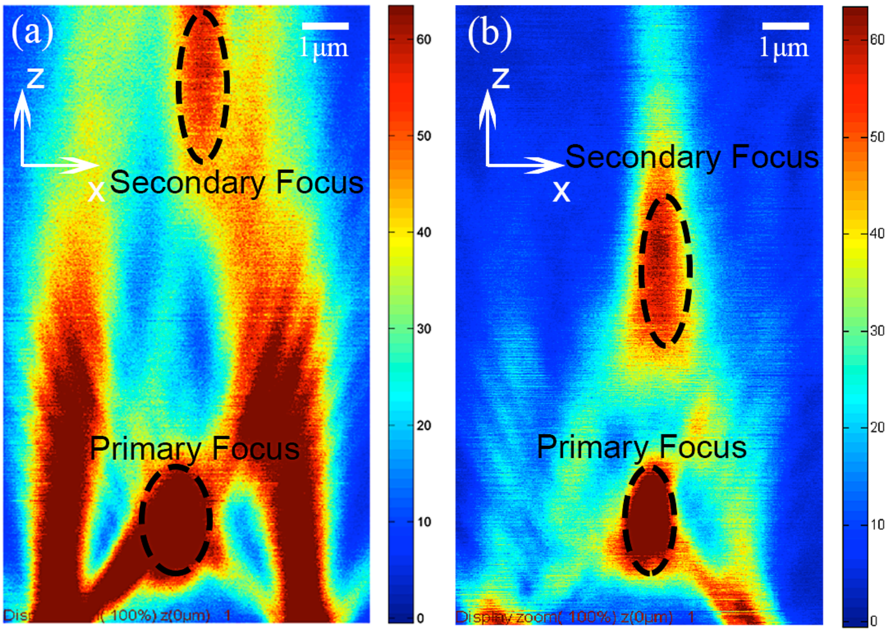

Supplementary Figure S2 | Transmission field characteristics. Experimental intensity distributions of the transmission field of the PASL structure under (a) 532 nm and (d) 633 nm illumination, acquired via a Z-depth scan module (9×15 μm^2). The bottom of the picture is the sample plane. Two focal spots has been marked in black dashed circles.

To further prove the assumption mentioned above, we also detected the polarization states of the secondary focal spot. When the incident polarization changes, the intensity distributions of the secondary focal spots in the two circular polarization situations show almost no differences (see Figure S3, (b) and (g)). The intensity of the

focal spot changes (see grey-scale value in Fig.S3(c-f), (h-k)) in the same manner as the incident light when the analyser is rotated. So we can attribute the intensity variation to the imperfect incident polarization and the low dynamic luminosity range of the CCD detector used.

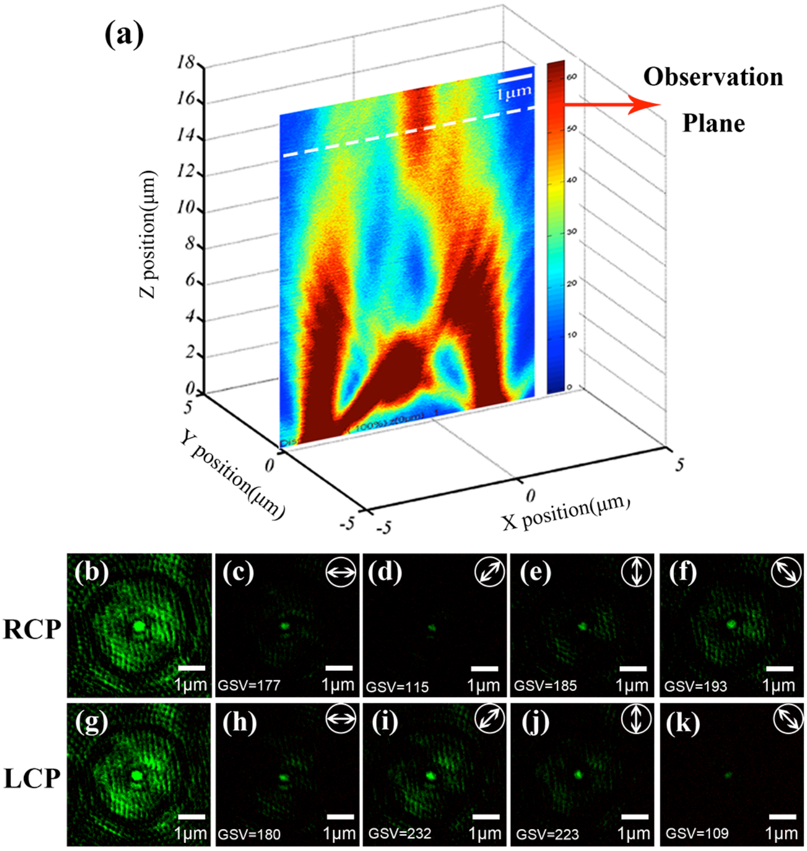

Supplementary Figure S3 | Intensity distributions in the two different circular polarization excitation cases. (a) The observation plane was placed in the second focal plane by the Z-depth scan module. The intensity distributions are shown for the (b) RCP and (g) LCP situations without the analyser. After insertion of the analyzer, the intensity distributions are shown for the (c)–(f) RCP and (h)–(k) LCP situations. The polarization orientation of the analyser is depicted using white arrows in the top-right corner of each picture. The GSV (grey-scale value) of central focal spot is marked in the bottom-left corner of each picture.
